# Laser performance and investigation of the optimal density functional and the dependence of the basis sets for (E, E)-2,5-bis (3,4-dimethoxystyryl) pyrazine (BDP) molecule

**DOI:** 10.1007/s00894-021-04876-0

**Published:** 2021-08-20

**Authors:** Mahmoud A. S. Sakr, Sayed A. Abdel Gawad, Samy A. El-Daly, Maram T. H. Abou Kana, El-Zeiny M. Ebeid

**Affiliations:** 1 Chemistry Department, Faculty of Science, Tanta University, Tanta, Egypt; 2 Misr University for Science and Technology (MUST), 6Th of October City, Egypt; 3 Laser Systems Department, National Institute of Laser- Enhanced Science (NILES), Cairo University, Cairo, Egypt

**Keywords:** Sol–gel, Copolymer, Quantum dots (QDs), Laser behavior, Photostability, Time-dependent density function theory (TDDFT)

## Abstract

This manuscript includes some photophysical parameters and some optical properties such as absorption and emission spectra of the (E, E)-2,5-bis (3,4-dimethoxystyryl) pyrazine (BDP) by applying sol–gel and copolymer matrices. The BDP molecular structure is incorporated in sol–gel matrix and copolymer of methyl methacrylate (MMA) and 2-hydroxyethyl methacrylate (HEMA). In case of sol–gel matrix, the BDP molecular structure has higher quantum yield in addition to photostability maxima. The laser behavior of this molecular structure containing sol–gel matrix is good senior compared to copolymer one via using diode laser (450 nm) as pumping laser of power 160 mW. Also, the fluorescence profile of the BDP molecular structure is sensitized via using cadmium sulfide (CdS) quantum dots (QDs) by applying sol–gel host. The optimized structure of the BDP molecule is obtained via applying B3LYP/6-31G(d) level of theory. The electronic absorption and emission spectra of the BDP molecular structure in ethanol solvent were calculated using time-dependent density functional theory (TDDFT) at CAM-B3LYP/6-31G +  + (d, p) level. The obtained theoretical results were compared to experimental ones.

## Introduction

In our previous work, some photophysical parameters, laser performance, quantum dot photosensitization, and TDDFT of (E, E)-2,5-bis [2-(4-(dimethylamino)phenyl) ethenyl]pyrazine (BDPEP) molecular structure via applying sol–gel and copolymer matrices are studied [1]. Consequently, the same previous studies for BDP molecular structure are investigated due to the different chemical structure of BDP molecule compared to BDPEP molecular structure.

As of late, a great deal of a work is presented to actualize laser dyes via applying solid-state as a useful option in contrast to the partner of liquid dye lasers owing to their compactness, ease of manufacture, low cost of fabrication, simplicity, and well-being in taking care of and activity, in addition to avoiding lack of toxicity, flammability, flow fluctuations, and solvent evaporation problems [2–4]. A lot of laser dyes via applying solid hosts have been broadly announced [5–15]. In solid-state dye lasers (SDDL) [16], the dye is interacted with matrix through hydrogen bonds among the dye and hydroxyls. This allows the dye to be adsorbed on the surface of the matrix, thereby preventing the dye bimolecular reactions and upgrading the dye thermal and environmental stability [17, 18].

The gain region was tailored over a wide range through the self-modulation of the optically activated ICT isomers. Meanwhile, the resonant modes shifted with the photoisomerization because of a change in the effective refractive index of the polymer microdisk cavity [19]. Polystyrene (PS) and poly(methylmethacrylate) (PMMA), with different polarities, were selected as the matrix materials to create Janus microlasers. The Janus structures were obtained by inducing phase separation of PS/ PMMA within the micrometer-sized emulsion droplets [20]. Laser displays, benefiting from the characteristic merits of lasers, have led to the revolution of next-generation display technologies owing to their superior color expression. However, the acquisition of pixelated laser arrays as self-emissive panels for flat-panel laser displays remains challenging. Liquid crystal (LC) materials with excellent processability and optoelectronic properties offer considerable potential for the construction of highly ordered multicolor laser arrays [21]. Miniaturized lasers with multicolor output and high spectral purity are indispensable for various ultracompact photonic devices. Here, we propose an optically reconfigurable Förster resonance energy transfer (FRET) process to realize broadband switchable single-mode lasing based on in situ activation of acceptors [22].

In a prior investigation, we detailed the laser activity and optical behavior of the BDP laser dyes in organic solvents [23]. Hence, the study the performance of this laser dye via applying sol–gel matrix and copolymer media is essential to upgrade the nature of laser action. The existence of HEMA kept up the transparency of solid matrix as well as ensured a good solubility of probe dye because of the polar character of HEMA [24, 25]. In the preparation of copolymer (MMA and HEMA), azobisisobutyronitrile (AIBN) was used as an initiator for free radical bulk polymerization of HEMA and MMA. The optical properties and lasing action of the BDP molecule via applying sol–gel and copolymer matrices are assessed.

Organic particles with huge delocalized л-electron systems along their backbone have attracted significant interest because of potential applications related with strong emission behavior and enormous nonlinear optical properties [26–28]. The electron-donating and electron-withdrawing groups play important roles in the optical properties of organic compounds [29]. The six-membered ring that contains nitrogen atoms at the 1,4 positions is called pyrazines [29, 30]. Having highly electron-withdrawing character, pyrazine compounds are perfect for the incorporation as electron-withdrawing groups in favoring intramolecular charge transfer (ICT) [29].

Quantum dots (QDs) have large potential uses as photosensitizers for a variety of application. Upon resizing the QDs, the electronic absorption and emission spectra of it can be adjusted. Studies recording the factors controlling the potential energy transfer process among donor molecule (QD) and acceptor molecules are investigated [31].

## Materials and methods

### Materials preparation

#### The BDP molecule preparation [32]

**Scheme 1:**
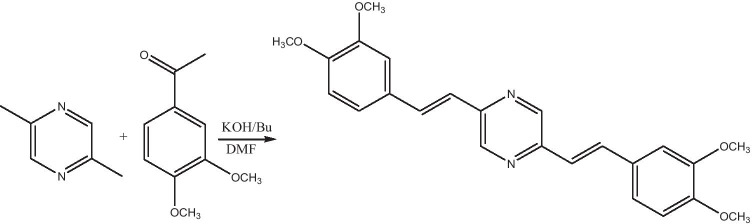
Reaction progress of preparation of BDP molecule

In dimethylformamide (DMF), the mixture from 2,5-dimethylpyrazine(0.5 g, 0.00462 M) and 3,4-dimethoxybenzaldhyde (2.3 g, 0.013 M) were dissolved and the mixture were cooled to 0.0 °C (Scheme 1). Then, potassium-tert-butanolate (1.55 g, 13 mM) was added with small amount to this mixture and brought to ambient temperature. Via applying stirrer, the mixture was stirred till completely consumed. By extraction with chloroform, the product was isolated after the completion the reaction. The product material obtained was recrystallized to give (1.4 g, 52%) as a yellow solid. The structure of the compound was assured via applying^1^H NMR and ^13^C NMR.

#### The preparation sol–gel matrix containing the BDP molecule [32]

**Scheme 2:**
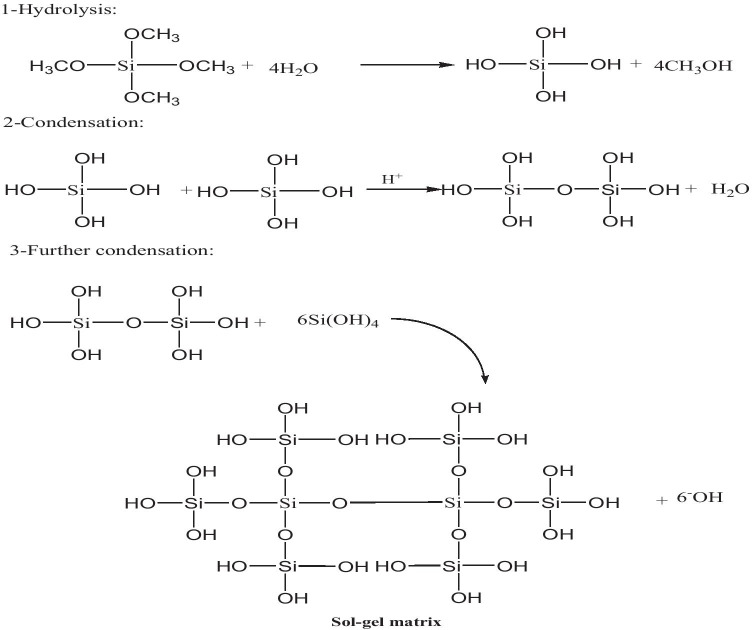
Preparation procedures for sol–gel matrix

The sol–gel matrix was prepared as follows (Scheme 2): via applying the magnetic stirrer, we mix (11.00 mL tetraethoxysilane (TEOS) (Aldrich, 98%), 6.00 mL methanol (Aldrich), 9.00 mL distilled water, 1 mL of hydrochloric acid (HCl) (Merck, approximately 35% pure, 1.18 gm^−1^) (0.1 N) as a catalyst, and 8.00 mL glycerol (Merck, IP for analysis) as a drying control chemical (DCCA) for 6 h at 60 °C. In polystyrene cuvette, 1.00 mL of the BDP solution is mixed with 3.00 mL sol. It was dehydrated and aging for about 3 weeks via applying a controlled oven at 60 °C.

#### The preparation of sol–gel matrix containing CdS QDs [33]

In order to prepare QDs in the silica matrix, QDs should be synthesized in aqueous rather than organic medium, as the solubility of aqueous QDs in sol–gel is excellent compared to organic QDs. Thus, CdS QDs in aqueous media is prepared as follows: 250 mL three-necked flask. In this flask, we mixed 100 mL CdCl_2_ (0.02 M), 2.5 mL H_2_O, then under stirring, 1.0 mL from thioglycolic acid was added. The PH was fixed to 12.00 via using 1.00 M NaOH. Then, 0.3 mL of 30% H_2_O_2_ drop wisely added in the flask after moving the solution for 10 min.

#### The preparation of copolymer (MMA/ HEMA) containing BDP molecule [34]

The copolymer of methylmethacrylate (MMA) and hydroxyethyl methacrylate (HEMA) was prepared via mixing MMA and HEMA (volume ratio 2:1) monomers containing the initiator azobisisobutyronitrile (AIBN) 3 g/L dissolved in it. Then, the prepared solution was subjected to ultrasonic stirring for about 15 min until the initiator was completely dissolved. After that, the BDP solution was added to obtain the desired concentration and allowed to dissolve in the ultrasonic bath for up to 20 min. In controlled clean oven at 60 °C as an optimum temperature regarding physical appearance and transparency, drying and aging were carried out. After 1 week from the date of preparation, the samples got dried so they could be handled and subjected to various measurements.

### Characterization

Via applying a transmission electron microscope (TEM) with acceleration voltage up to 200 kV, the morphology and the size of the CdS QDs were recorded. Utilizing UV–Vis spectrophotometer (Shimadzu UV-2450), the optical properties of the BDP molecule are measured over a range of 200–700 nm. The fluorescence properties were measured via utilizing spectrofluorometer (Shimadzu, RF-5301PC).

## Results and discussion

**Fig. 1:**
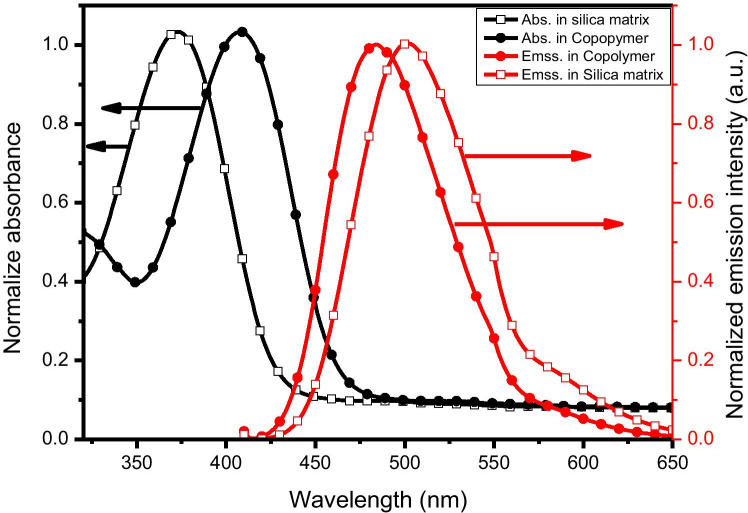
Normalized absorption (in black) and emission (in red) spectra of 1 × 10^–5^ M BDP in sol–gel and copolymer matrices

The electronic absorption and emission spectra of the studied molecule was measured experimentally via applying sol–gel and copolymer matrices; the results are presented in Fig. 1. The electronic absorption spectrum of the studied molecular structure in sol–gel and copolymer matrices consists of mainly one peak appearing at 374 and 409 nm, respectively. It is assigned as π-π* transition as indicated by its extinction coefficient, 8280 and 7250 Lmol^−1^ cm^−1^for the studied molecule via applying sol–gel and copolymer matrices, respectively. On the other hand, the electronic emission spectra of the studied system were also recorded by applying sol–gel and copolymer matrices and presented in Fig. 1. The experimental maximum emission wavelength of the BDP molecular structure in sol–gel and copolymer matrices is 502 and 485 nm, respectively. As presented in Fig. 1, the interference among the experimental electronic absorption and emission spectra were not observed by applying sol–gel contrast to copolymer one. Therefore, the reabsorption of the experimental electronic emission photons of the studied molecular structure has not been occurred by applying sol–gel matrix contrast to copolymer host as presented in Fig. 1.

Via utilizing Fig. 1, the fluorescence quantum yield (ϕ_f_) [34, 35], oscillator strength ($f$), transition dipole moment from ground to excited state (μ_12_) [36, 37], and radiative decay rate constant (K_r_) [38] for the studied molecule by applying sol–gel and copolymer matrices are estimated and presented in Table 1. The ϕ_f_ value of the studied molecule by applying sol–gel matrix is high in contrast to copolymer matrix as listed in Table 1, owing to high mobility of the BDP molecules in sol–gel compared to copolymer host. In addition, the highest value of K_r_ of the BDP molecule embedded sol–gel matrix in contrast to copolymer one as shown in Table 1. Referring to the high singlet–triplet splitting energies (ΔE_S,T_) of the studied molecular structure by applying sol–gel matrix compared to the copolymer host [39–42]. The value of *f* and μ_12_ of the studied molecule via applying sol–gel and copolymer matrices indicating (S_0_ to S_1_ electronic transition).

**Table 1:** Photo physical parameters of BDP molecule by applying sol–gel and copolymer matrices

Hosts	λ_abs_. (nm)	λ_ems_ (nm)	ϕ_f_	$f$	K_r_ × 10^9^ (S^−1^)	μ_12_ (Debay)
Sol–gel	374	502	0.52	0.82	0.85	9.63
Copolymer	409	485	0.43	0.62	0.79	8.62

**Fig. 2:**
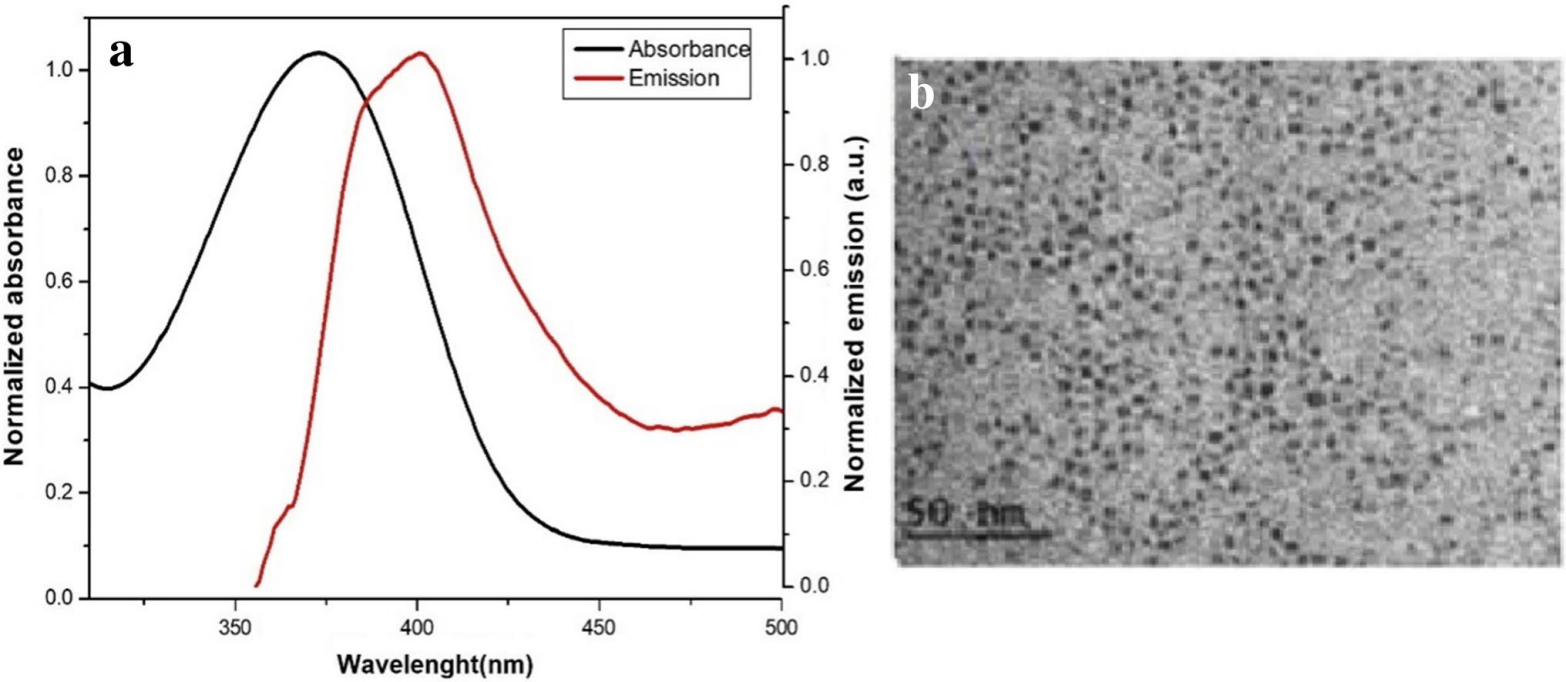
**a** The spectral overlaps of 1 × 10^–5^ M of BDP absorption and 6.6 × 10^–6^ mol/L CdS QDs emission spectra (excitation wavelength λ_ex._ = 380 nm) in silica matrix. **b** The TEM image of CdS QDs

The experimental overlapping between electronic absorption spectrum of the studied molecule and emission spectrum the CdS QDs by applying sol–gel matrix was examined; the results are given in Fig. 2a. Via applying the following equations [43],

$$D=\left(1.6122\times {10}^{-9}\right){\lambda }^{4}-\left(2.6575\times {10}^{-6}\right){\lambda }^{3}+\left(1.6242\times {10}^{-3}\right){\lambda }^{2}-\left(0.4277\right)\lambda +\left(41.57\right)$$

_,_

$$\varepsilon =5857{\left(D\right)}^{2.65}$$,

where *D* (nm) is the size of the nanocrystal administration sample, $\lambda$ (nm) is the wavelength, and $\varepsilon$ is the molar absorptivity of the first excitonic absorption peak of the corresponding sample. The used molar concentration of the synthesized CdS QDs was 6.6 × 10^–6^ M and the CdS QDs size was 3–6 nm. The CdS QDs TEM image is shown in Fig. 2b.

**Fig. 3:**
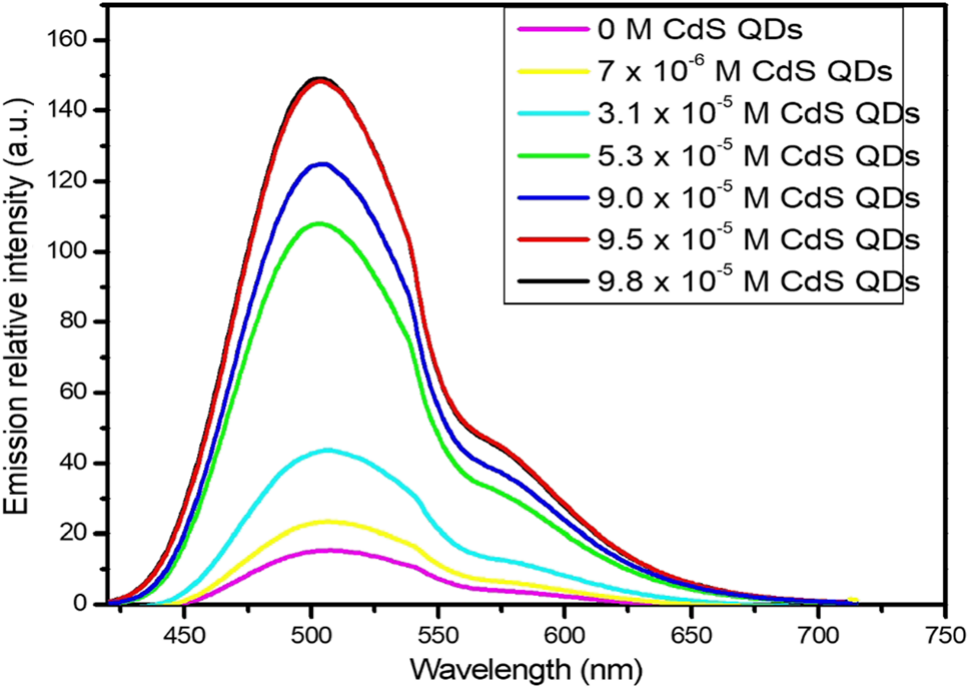
Emission spectra of 1 × 10^–5^ M BDP dye in sol–gel matrix at different concentrations of CdS QDs (excitation wavelength λ_ex._ = 380 nm)

Due to large overlapping between emission spectrum of the CdS QDs and absorption spectrum of the studied molecule by applying sol–gel matrix as presented in Fig. 2a. The emission spectrum of the studied molecule was upgraded in sol–gel matrix via adding different concentrations of CdS QDs as shown in Fig. 3. The experimental electronic maximum fluorescence intensity at 503 nm is enhanced subsequently and the position of the electronic maximum emission wavelength at 503 nm is not influenced as shown in Fig. 3.This refers to the fluorescence emission of the studied molecule in sol–gel matrix which is photosensitized via applying different concentration of CdS QDs.

**Fig. 4:**
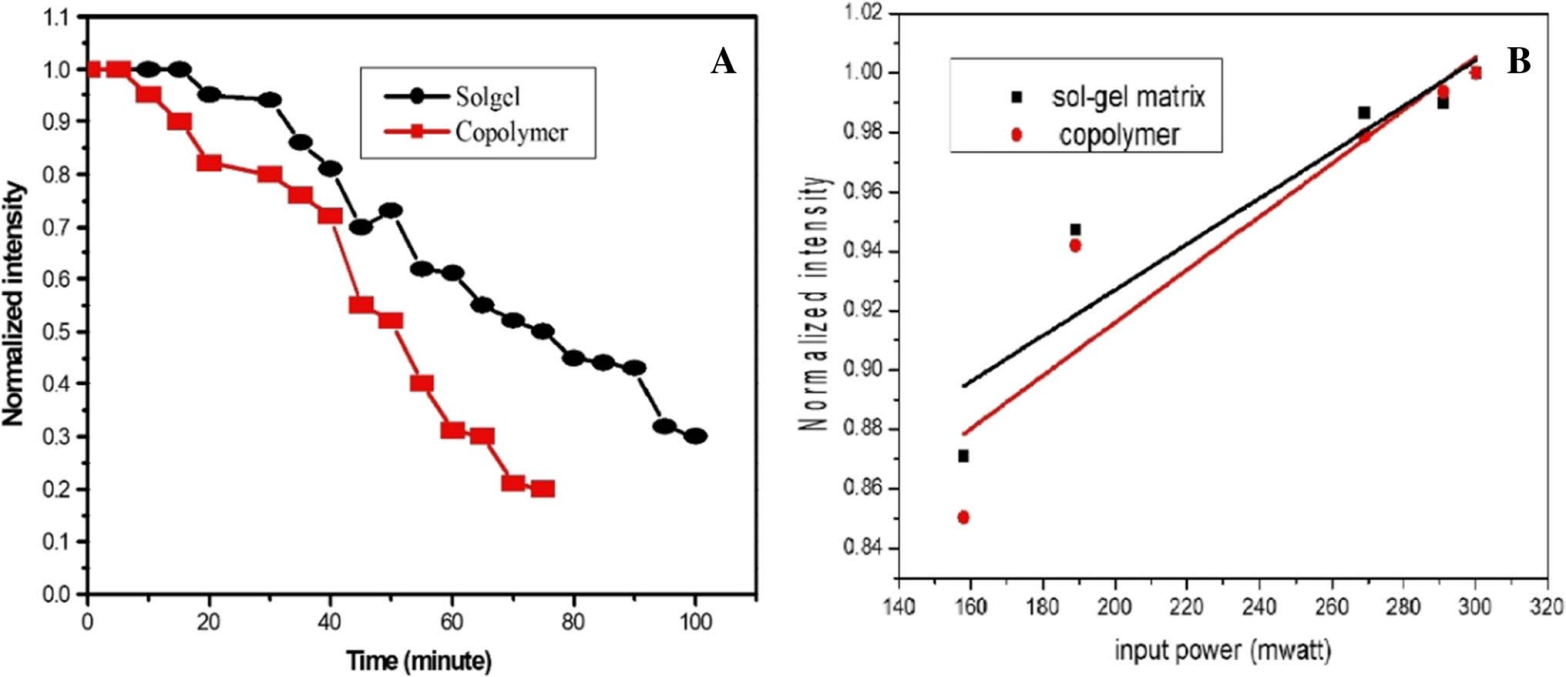
The output intensity as a function of time utilizing a 450-nm wavelength diode laser and a power of 160 mW as a source of pumping (**A**) and the output intensity as a function of different input energy (**B**) for 1 × 10^–3^ M BDP dye by applying both sol–gel and copolymer matrices utilizing diode continuous laser (λ = 450 nm)

Assume that the photo-stability of the sample is defined as the time needed to reduce the energy output intensity to half of its value. Hence, the half-life time of the BDP molecular structure embedded sol–gel and copolymer matrices is 70 and 50 min, respectively, as shown in Fig. 4A using a 450-nm wavelength diode laser and a power of 160 mW as a source of pumping. Consequently, the half-life time of the BDP molecular structure via applying sol–gel matrix is higher than that copolymer because the mobility of the BDP molecules in sol–gel matrix is higher than that in copolymer.

Suppose that the amplified spontaneous emission (ASE) efficiency is defined as the ratio between the output energy of the target molecule over arrange of input pumping energy [44]. Hence, the ASE (a.u.) efficiency of 1 × 10^–3^ M BDP molecule via applying sol–gel matrix is higher than that in copolymer as given in Fig. 4B, which indicates the highest number of excited BDP molecules in sol–gel matrix in contrast to copolymer one. Via utilizing diode laser of 450-nm wavelength, the input energy was controlled and varied between 160 and 300 mW.

**Fig. 5:**
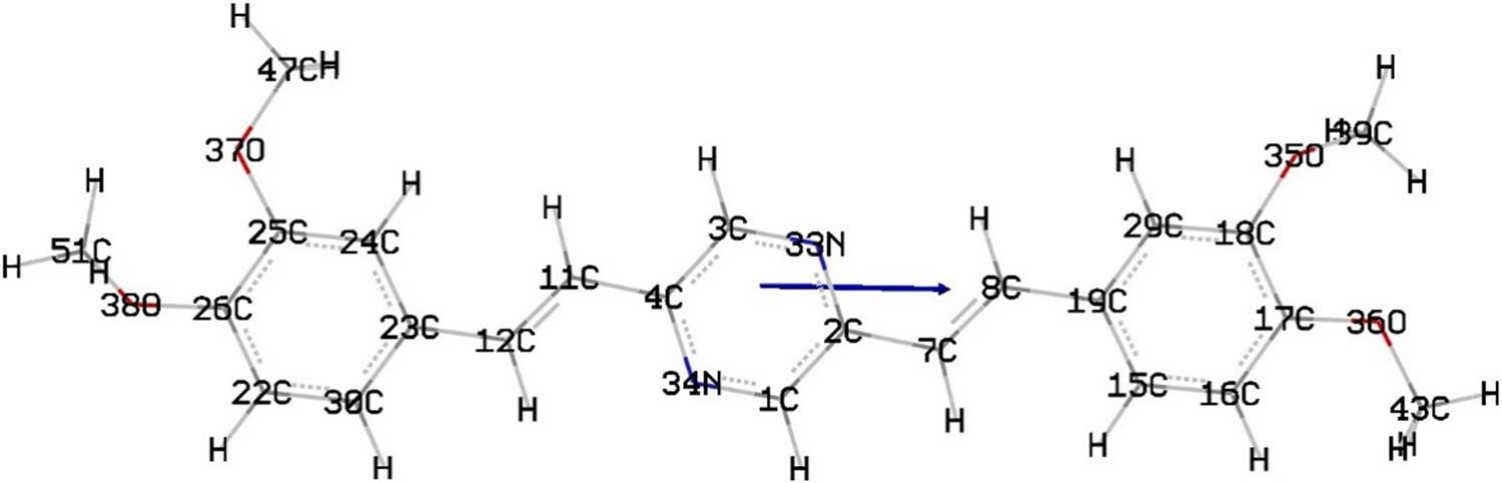
Optimized geom. of compound

The electronic ground-state structures of the studied compounds were obtained utilizing the density functional theory (DFT) [45] method. The chemical structure optimizations were carried out at the [46, 47] B3LYP/6-31G(d) level of theory and the results are given in Fig. 5. BDP molecular structure planar is presented in Fig. 5. The geometrical parameters of the BDP compound in gas state are listed in Table 2. The labeling scheme is recorded in Fig. 5. Table 2 showed some selected bond lengths and dihedral angles of the studied BDP molecular structure. From Table 2, some comments can be listed: (1) referring to the listed dihedral angles, the two phenyl rings and the pyrazine ring together with the two vinyl groups all lie in the same plane. (2) The bond lengths of the two vinyl groups (C_12_-C_11_ and C_8_-C_7_) are very slightly different (ca. 0.001 Å). (3) The two methyl groups that bind to O_38_ and O_35_ rotate by dihedral angle 110.78 and 108.97° due to steric hindrance.(4) The obtained bond angles refers to the sp^2^ hybridization over the entire molecular structure. (5) The changes in the obtained bond lengths as all carbon–carbon, ring’s carbon–oxygen, and ring’s carbon–nitrogen bonds are either doubly bonded or partially multiply bonded.

**Table 2:** Selected optimized geometrical parameters (bond length in Å and dihedral angle in °) computed for BDP molecule in gas phase using B3LYP/6-31G(d). For labeling, refer to Fig. 5

C26-O38	1.373	C47-O37-C25-C26	179.77
C25-O37	1.363	C51-O38-C26-C22	110.78
C23-C12	1.461	C3-N33-C2-C7	179.79
C12-C11	1.351	C39-O35-C18-C29	108.97
C11-C4	1.457	C43-O36-C17-C18	179.61
C2-C7	1.456	C16-C17-O36	124.89
C7-C8	1.352	O35-C18-C17	120.85
C8-C19	1.459	O37-C25-C26	115.69
C17-O36	1.359	O38-C26-C25	121.11
C18-O35	1.377	C12-C11-C4	124.11

**Fig. 6:**
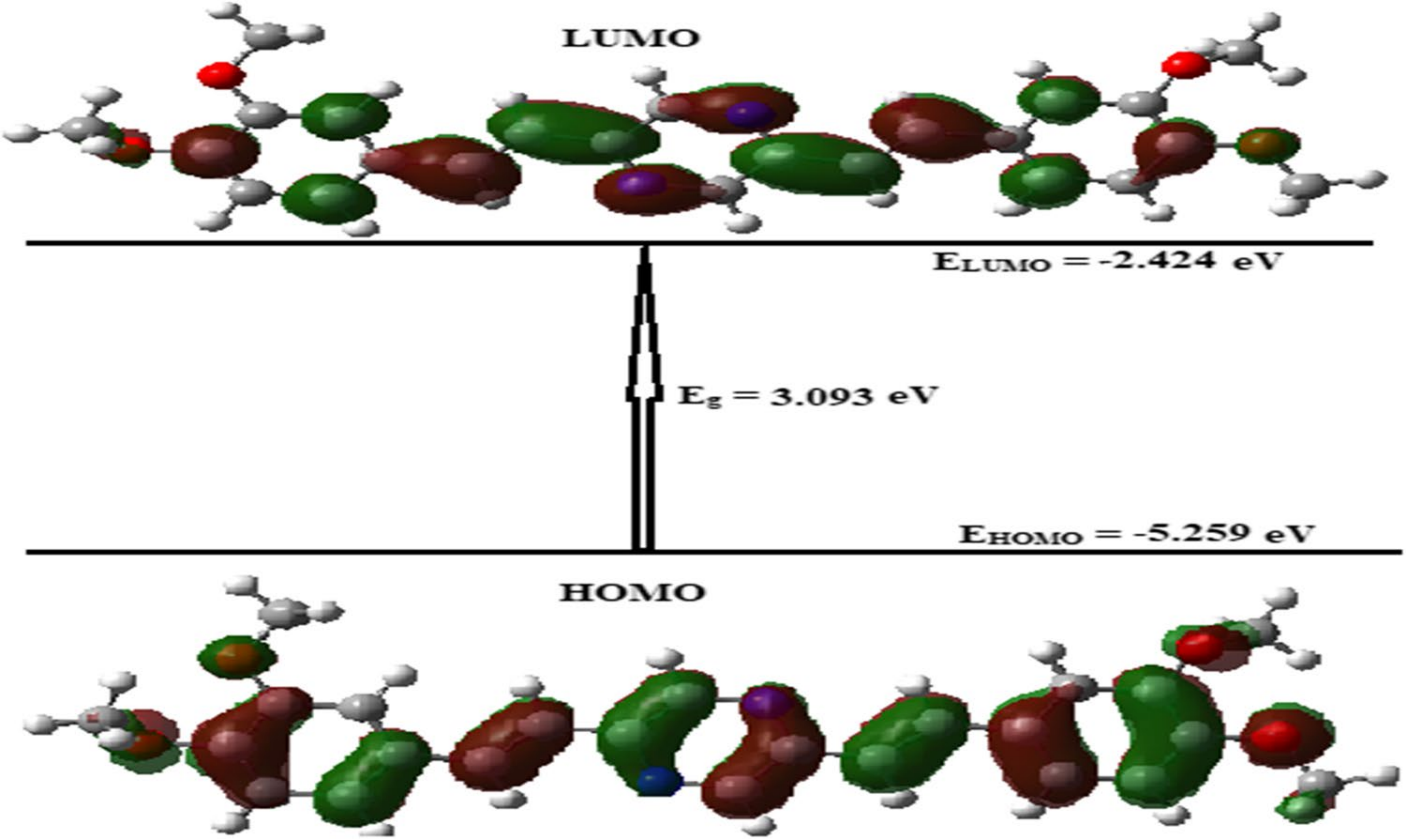
The graphical presentation of the highest occupied (HOMO) and lowest unoccupied molecular (LUMO) orbitals of BDP molecular structure in gas via utilizing B3LYB/6-31G(d) level of theory

The graphical presentation of the HOMO/LUMO orbitals and energy gap between HOMO and LUMO (E_g_) for BDP compound in gas at B3LYB/6-31G(d) level of theory is shown in Fig. 6. The л-bonding orbitals and the lone pairs of electrons of the oxygen atoms of the BDP molecule are delocalized over the whole BDP molecule(see Fig. 6). In addition to, the л* anti-bonding orbitals in the LUMO energy level (see Fig. 6) are distributed over the whole of the BDP molecular structure, while the four oxygen atoms in the BDP molecule have a small part in distribution over the whole molecule.

**Fig. 7:**
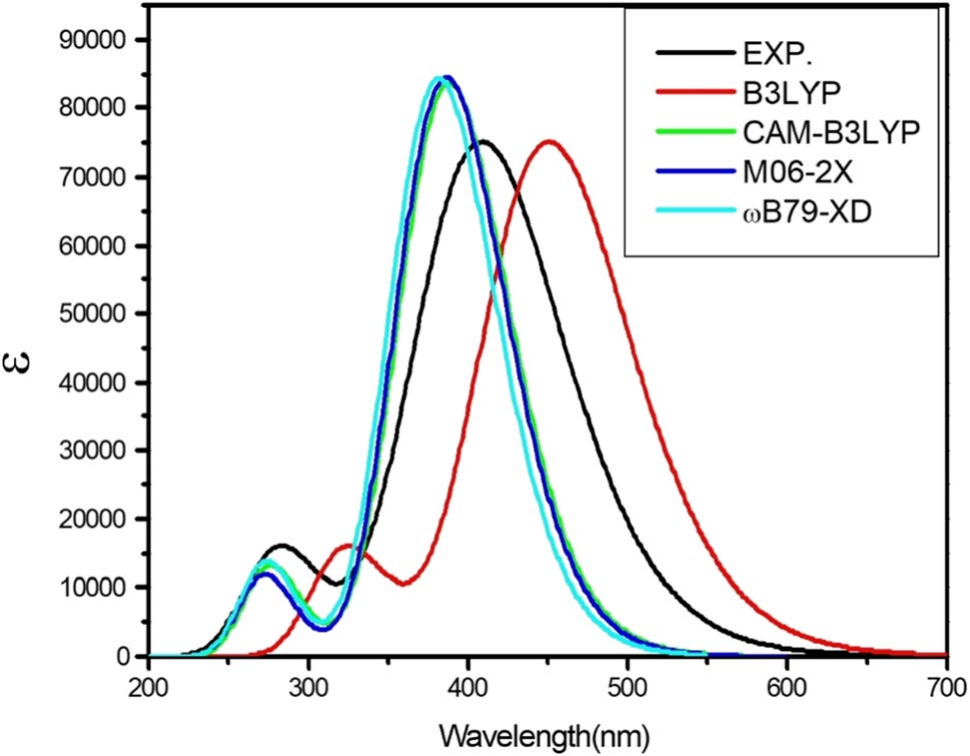
Calculated absorption spectra of BDP molecular structure given with the utilization of various functionals, B3LYP, CAMB3LYP, M06-2X, and ωB97X-D and experimental absorption spectrum of the BDP (in black). Y-axis refers to calculated absorptivity. The 6-31G(d) basis set was satisfied

**Fig. 8:**
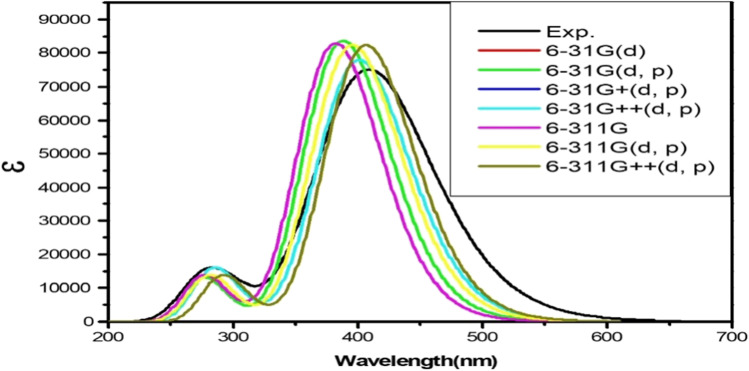
Calculated electronic absorption spectra of the BDP in ethanol with different basis sets and practical absorption spectrum (in black).Y-axis refers to calculated absorptivity. The CAM-B3LYPfunctional was applied

The optimal density functional and the dependence of the basis sets for BDP molecular structure are investigated in two steps; the results are presented in Figs. 7 and 8. In the first step, the choice of the optimal dependence of the DFT functionals are studied (B3LYP [48], CAM-B3LYP[49], M06-2X [50], ωB97X-D [51]) [52] as recorded in Fig. 7. The optimal DFT function for calculated electronic absorption spectra of the BDP molecular structure in ethanol is CAM-B3LYP (see Fig. 7). Via applying CAM-B3LYP function, the calculated electronic absorption spectrum of the BDP molecular structure is in agreement with the experimental results as recorded in Fig. 7.

In the second step, the dependence on the basis set is recorded via applying the CAM-B3LYP functional. The computational electronic absorption spectra of the BDP molecular structure in ethanol were studied by applying different basis sets; the results are given in Fig. 8. The diffuse functions must be used to obtain the more accurate electronic absorption results for BDP molecular structure in ethanol solvent as presented in Table 3. Therefore, the optical properties of the BDP molecular structure in ethanol are investigated via applying CAM-B3LYP functional with the 6–311 +  + G (d, p) basis set.

**Table 3:** The calculated maximum absorption wavelength (Calc. λ_abs_. (nm)) of the BDP in ethanol with the use of different basis sets and practical absorption wavelength (Exp. λ_abs_. (nm)). The CAM-B3LYPfunctional was applied

Basis sets	Calc. λ_abs_. (nm)	Pract. λ_abs_. (nm)
6-31G(d)	388	408
6-31G(d, p)	388	
6-31G + (d, p)	401	
6-31G + + (d, p)	401	
6-311G	382	
6-311G(d, p)	395	
6-311G + + (d, p)	406	

**Fig. 9:**
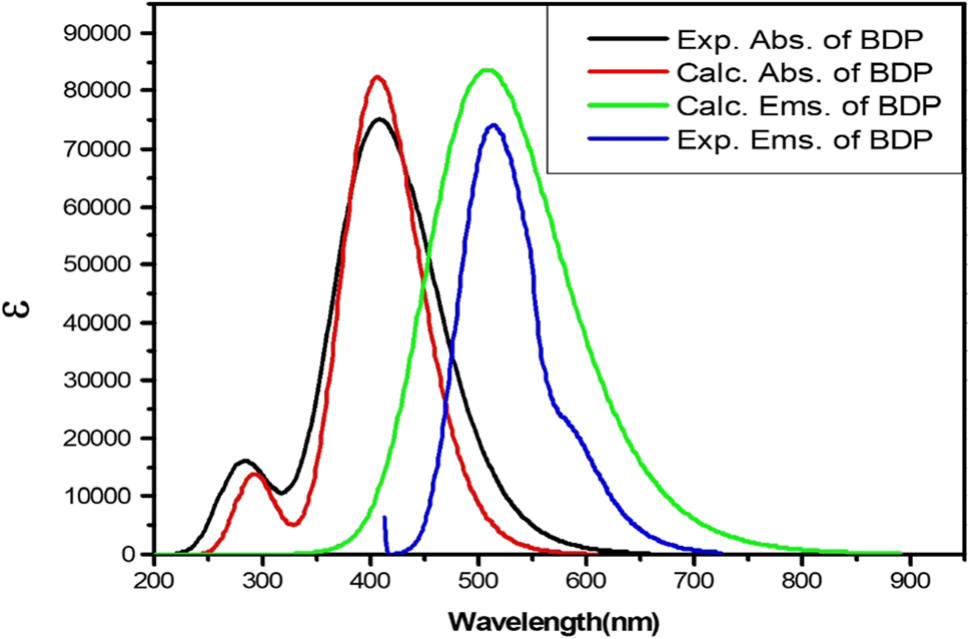
Calculated electronic absorption (Calc. Abs.) and emission spectra (Calc. Ems.) of the BDP in ethanol with different basis sets and experimental absorption (Exp. Abs.) and emission spectra (Exp. Ems.) of the BDP. Y-axis refers to calculated absorptivity. The CAM-B3LYP functional was applied with 6-311G +  + (d, p) basis set

The calculated and experimental electronic absorption and emission spectra of the BDP molecule in ethanol are studied; the obtained spectra are presented in Fig. 9. The calculated electronic maximum absorption and emission wavelength of the BDP molecule are 406 and 508 nm, respectively, as presented in Fig. 9. In addition, the experimental maximum absorption and emission wavelength of the BDP molecule are 408 and 514 nm, respectively, as presented in Fig. 9. Therefore, the computational calculated absorption and emission spectra of the BDP molecule are in agreement with experimental results as recorded in Fig. 9. From previous study and this study, the photophysical parameters, laser performance, and QDs photosensitization of the BDPEP and BDP molecular structures are upgraded by applying sol–gel matrix compared to copolymer. In addition, the calculated optical properties of the BDPEP and BDP molecular structures are in agreement with experimental results using M06-2X/6-311G +  + (d, p) andCAM-B3LYP/6–311 +  + (d, p) levels, respectively.

## Conclusion

The photophysical parameters such as fluorescence quantum yield (Φ_f_), oscillator strength ($f$), radiative decay rate constant (K_r_), change dipole moment from ground to excited state (μ_12_), gain coefficient, and efficiency of the BDP molecule via applying sol–gel matrix are upgraded compared to copolymer. The mixing of CdS QDs to the BDP compound in sol–gel matrix is obtained successfully. The fluorescence emission of the BDP upon using sol–gel matrix is sensitized via adding different concentration of the CdS QDs. The lasing properties and photostability of the BDP molecule via applying sol–gel are recorded and compared to copolymer matrices. From these studies, we concluded that the photodegradation rates for the BDP molecular structure via utilizing sol–gel matrix was generally lower than via using copolymer. The optical properties of the BDP molecule are calculated using the CAM-B3LYPfunctional with the 6–311 +  + G(d, p) basis set. The computational calculated absorption and emission spectra of the BDP molecule are in agreement with experimental results.

## Data Availability

All data generated or analyzed during this study are included in this published article.

## Code availability

N/A.
